# Supplementary material for: The impact of the SARS-CoV-2 pandemic on the demographic, clinical and social profiles of patients admitted to the Pneumology Department for a COPD exacerbation
Source: PLoS One. 2023 Sep 14;18(9):e0290156. doi: 10.1371/journal.pone.0290156 (PMC10501653; doi:10.1371/journal.pone.0290156)
Supplement: Attachment — Submitted filename: Response to Reviewers.docx [file pone.0290156.s002.docx]

**1. Please ensure that your manuscript meets PLOS ONE's style requirements, including those for file naming.**

Appropriate adjustments have been made.

**2. Please provide additional details regarding participant consent. In the ethics statement in the Methods and online submission information, please ensure that you have specified what type you obtained (for instance, written or verbal, and if verbal, how it was documented and witnessed). If your study included minors, state whether you obtained consent from parents or guardians. If the need for consent was waived by the ethics committee, please include this information.**

This study was approved by the Galician Research and Ethics Committee (ethical clearance number 2016/524) and all the patients signed their written informed consent to participation.

This information has been included in the manuscript.

**3. We note that you have included the phrase “data not shown” in your manuscript. Unfortunately, this does not meet our data sharing requirements. PLOS does not permit references to inaccessible data. We require that authors provide all relevant data within the paper, Supporting Information files, or in an acceptable, public repository. Please add a citation to support this phrase or upload the data that corresponds with these findings to a stable repository (such as Figshare or Dryad) and provide and URLs, DOIs, or accession numbers that may be used to access these data. Or, if the data are not a core part of the research being presented in your study, we ask that you remove the phrase that refers to these data.**

"Data not shown" refers in this case to the fact that the results are not detailed in the included table but are results derived from the study itself and described in the manuscript. To avoid this confusion, the phrase was deleted.

**4. Thank you for stating the following in the Competing Interests section:**

**“None of the authors declare conflicts of interest for the contents of this manuscript.**

**AFV has received honoraria in the last 3 years for lecturing, scientific consulting, participating in clinical studies, or writing publications for (alphabetical order): AstraZeneca, Boehringer Ingelheim, Chiesi, GlaxoSmithKline, Grifols, and Menarini.**

**CRR has received honoraria in the past 3 years for lecturing, scientific consulting, clinical trial participation, or publication writing for (alphabetical order): AstraZeneca, Boehringer Ingelheim, Chiesi, Faes farma, and GlaxoSmithKline.”**

**Please confirm that this does not alter your adherence to all PLOS ONE policies on sharing data and materials, by including the following statement: "This does not alter our adherence to  PLOS ONE policies on sharing data and materials.” (as detailed online in our guide for authors http://journals.plos.org/plosone/s/competing-interests).  If there are restrictions on sharing of data and/or materials, please state these. Please note that we cannot proceed with consideration of your article until this information has been declared.**

**Please include your updated Competing Interests statement in your cover letter; we will change the online submission form on your behalf.**

The indicated sentence has been included. The conflict of interest statement has been included on the cover page.

**5. Please include your full ethics statement in the ‘Methods’ section of your manuscript file. In your statement, please include the full name of the IRB or ethics committee who approved or waived your study, as well as whether or not you obtained informed written or verbal consent. If consent was waived for your study, please include this information in your statement as well.**

Full ethics statement has been included in the Methods section.

**6. Please include captions for your Supporting Information files at the end of your manuscript, and update any in-text citations to match accordingly. Please see our Supporting Information guidelines for more information:** [**http://journals.plos.org/plosone/s/supporting-information**](http://journals.plos.org/plosone/s/supporting-information)**.**

Appropriate changes have been made to supplementary material.

**7. In your Data Availability statement, you have not specified where the minimal data set underlying the results described in your manuscript can be found. PLOS defines a study's minimal data set as the underlying data used to reach the conclusions drawn in the manuscript and any additional data required to replicate the reported study findings in their entirety. All PLOS journals require that the minimal data set be made fully available. For more information about our data policy, please see** [**http://journals.plos.org/plosone/s/data-availability**](http://journals.plos.org/plosone/s/data-availability)**.**

**Upon re-submitting your revised manuscript, please upload your study’s minimal underlying data set as either Supporting Information files or to a stable, public repository and include the relevant URLs, DOIs, or accession numbers within your revised cover letter. For a list of acceptable repositories, please see** [**http://journals.plos.org/plosone/s/data-availability#loc-recommended-repositories**](http://journals.plos.org/plosone/s/data-availability#loc-recommended-repositories)**. Any potentially identifying patient information must be fully anonymized.**

**Important: If there are ethical or legal restrictions to sharing your data publicly, please explain these restrictions in detail. Please see our guidelines for more information on what we consider unacceptable restrictions to publicly sharing data:** [**http://journals.plos.org/plosone/s/data-availability#loc-unacceptable-data-access-restrictions**](http://journals.plos.org/plosone/s/data-availability#loc-unacceptable-data-access-restrictions)**. Note that it is not acceptable for the authors to be the sole named individuals responsible for ensuring data access.**

**We will update your Data Availability statement to reflect the information you provide in your cover letter.**

These data comes from an approved study by the Committee of Ethics in Research. Our clinical data cannot be shared in a public repository because, even anonimized, the information could still identify the patients' identity using information such as sex, age, and place of residence (i.e. urban rural). All this information combined could lead to identifying patients and we were warned regarding this possibility when our research was authorized. However, in the event that new data on the study is required, the authors are willing to solve this information.

**8. Please review your reference list to ensure that it is complete and correct. If you have cited papers that have been retracted, please include the rationale for doing so in the manuscript text, or remove these references and replace them with relevant current references. Any changes to the reference list should be mentioned in the rebuttal letter that accompanies your revised manuscript. If you need to cite a retracted article, indicate the article’s retracted status in the References list and also include a citation and full reference for the retraction notice.**

References have been reviewed, no changes.

**Please rewrite the title according to the journal requirement**🡪 Done.

**The word AEPOC should be written in complete form in line 45**🡪 Done.

**In line 49, you used AECOPD, which also needs to be written in complete form**. 🡪 Done

**Line 47 seems grammatically incorrect. 🡪** Reviewed**.**

**Line 51 started with 340; a line should not start with a number.** 🡪 Modified.

**Line-105, please provide the ethical clearance number** 🡪 Done.

**Please give a patient selection flow chart in the result section**. 🡪 Patients were included **consecutively** following the inclusion and exclusion criteria described in the manuscript.

**Line 147 needs correction**. 🡪 Done.

**Please provide the actual results figure and p-value within the description**. 🡪 Done

**Give a result summary at the beginning of the discussion** 🡪 Done and highlighted in the text.

**Define some variables in the legends of the tables.** 🡪 Done.

**Please copy edit the manuscript for grammatical error**. 🡪 Reviewed.

**Answer to Reviewer #3:**

**I would only like to ask the authors whether the potential systemic manifestations of these patients were analyzed during the study period and whether any differences could be analyzed or at least envisaged. I would like to ask about whether these results could be extrapolated to other chronic airway diseases such as asthma or bronchiectasis.**

Regarding systemic manifestations, if the question refers to non-respiratory manifestations derived from COPD such as osteoporosis or loss of muscle mass, unfortunately these variables were not collected during the study, although their contribution would be interesting. However, the comorbidities included in the Charlson index were collected, without evidence of significant differences in the analysis performed.

With respect to other entities such as asthma or bronchiectasis, it is possible that the health and social measures derived from the pandemic that induced the changes described in patients with COPD, affected patients with other respiratory diseases in a similar way, especially those that present with infectious or inflammatory exacerbations. However, the results cannot be extrapolated since the study did not include patients with these characteristics, only patients with COPD, and there may be differences in the clinical profile (such as age, comorbidities...).

**Answers to Reviewer #4:** 

**1) How many of the patients had PAH on echo and how many of them presented with type 2 respiratory failure at the time of admission? How many had high PaCO2 and high bicarbonate at the time of discharge because these are again independent factors for readmission.**

Pulmonary arterial hypertension or pO2 and pCO2 values were not collected as variables for this study, since not all patients had an echocardiogram or blood gas at the time of hospital admission and discharge. Nonetheless, the need for oxygen therapy or non-invasive ventilation at discharge was collected as variables, which may be an indirect reflection of these values. However, the fact of needing any of these respiratory support therapies at discharge did not imply an independent risk factor for readmission in the analysis (p adjusted = 0.56).

**2) Did any of the patients also have associated LV failure or atrial fibrillation or multi focal atrial tachycardia and could these be important risk factors for readmission?**

I appreciate the question, I find it very interesting for the purpose of this study. In this case the history of heart failure and atrial fibrillation were collected, among other comorbidities. None of the comorbidities collected was shown to be a risk factor for readmissions. The component of heart failure or AF with uncontrolled ventricular response during the index admission was not analyzed. If considered necessary, it could be included as a limitation.

**3) The medication history is not mentioned in detail nor severity of the disease in terms of spirometry mentioned anywhere. Medication history in terms of high dose of inhaled steroids or even systemic steroids could have had a significant impact on the study.**

Regarding severity in terms of spirometry, no differences were found in readmissions according to the FEV1 value as described in the manuscript (p=0.91). Similarly, the variable triple therapy at discharge was collected as another treatment-related severity variable, this not being an independent risk factor for readmissions (adjusted p = 0.56). Treatment with systemic corticosteroids was not collected as a variable. However, the history of admission due to a severe COPD exacerbation in the previous year was collected, where the patient would foreseeably have received treatment with systemic corticosteroids following current treatment guidelines. This variable was not an independent risk factor either (p=0.55).

**4) What percentage of patients were involved in a pulmonary rehabilitation programme as that could have had a beneficial effect in preventing readmissions as also the influenza and pneumococcal vaccinations.**

During admission due to severe COPD exacerbation, all patients received respiratory physiotherapy as part of the severe exacerbation treatment program according to current guidelines. However, the data on which patients regularly participated in rehabilitation programs on an outpatient basis was not recorded, data that would probably be of interest. If the reviewer considers it necessary, it could be included as a limitation of the study. The vaccination advice is part of the health education for all patients with COPD under follow-up in the Pneumology Department. As described in the manuscript, what is interesting about this result is that, although the vaccination rate is considerable (greater than 65%), there is a significant increase in vaccination against influenza after the outbreak of another viral infection such as COVID-19, probably related to the fear factor associated with the infection.

**5) What were the various causes for AECOPD because some variables like a pneumonia, pneumothorax, structural disease as in terms of bronchiectasis, bullae could have had an impact on readmissions.**

The patient selection criteria for this study was hospital admission due to COPD exacerbation according to the criteria of the responsible physician defined by current clinical practice guidelines, excluding SARS COV2 infection. The presence of radiological infiltrates was one of the exclusion criteria for participation in the study, as well as patients with COPD where the reason for admission were independent entities such as pneumonia or pneumothorax, even though these could lead to an exacerbation of their COPD. The presence of bullae or bronchiectasis was not analyzed since not all patients had a CT at the time of admission.

**6) What was the level of self health care in both the groups and did both these groups have similar access to the care facility?**

The level of health self-care was not evaluated in a concrete way. However, one of the items valued in the instrumental activities is the patient's ability to manage their own medication. This item can indirectly assess their self-care in health. One of the differences evidenced is that the patients admitted during the pandemic were less dependent for instrumental activities (the management of their own medication, among others), both in the pre and post-vaccination phase. However, this variable was not a risk factor for readmissions.

Although the health system in which the study was carried out is public and universal, access to health care may depend on social factors such as those described (need for a caregiver, dependence for basic and instrumental activities, social relationships, living alone, rural residence, economic resources...). None of these criteria was a risk factor for readmission. Where differences were evident was in access to the health system during the pandemic. One of the conclusions of this study is that the difficulty in accessing non-urgent health care such as primary care or day hospitals probably precipitated an increased number of visits to the Emergency Department and readmissions, with the urgent route being in many cases the only way of access to the healthcare system during the pandemic.
