# Supplementary material for: The impact of the SARS-CoV-2 pandemic on the demographic, clinical and social profiles of patients admitted to the Pneumology Department for a COPD exacerbation
Source: PLoS One. 2023 Sep 14;18(9):e0290156. doi: 10.1371/journal.pone.0290156 (PMC10501653; doi:10.1371/journal.pone.0290156)
Supplement: Attachment — Submitted filename: Response to reviewers.docx [file pone.0290156.s004.docx]

**Journal Requirements:

Please review your reference list to ensure that it is complete and correct. If you have cited papers that have been retracted, please include the rationale for doing so in the manuscript text, or remove these references and replace them with relevant current references. Any changes to the reference list should be mentioned in the rebuttal letter that accompanies your revised manuscript. If you need to cite a retracted article, indicate the article’s retracted status in the References list and also include a citation and full reference for the retraction notice.**

Reviewed and corrected.

**Additional Editor Comments:

You've assigned roles to the authors in your manuscript, but not all of them met the criteria for authorship as outlined by the International Committee of Medical Journal Editors (ICMJE) Recommendations. The four criteria for authorship include substantial contributions to the conception, design, acquisition, analysis, or interpretation of data for the work; drafting or critically revising the work for important intellectual content; final approval of the version to be published; and agreement to be accountable for all aspects of the work.**

Reviewed and corrected.

**Additionally, your reference list contains 29 references, but you cited 30 in the text. Please ensure consistency between your reference list and citations.**

Reviewed and corrected.

**Lastly, please submit the figure file separately and include only the figure caption in the manuscript after it has been first cited. Refer to the Plos One instructions for submission of figures for guidance.**

Reviewed and corrected. Figure is attached in a separate file.
